# The Prediction Model of Risk Factors for COVID-19 Developing into Severe Illness Based on 1046 Patients with COVID-19

**DOI:** 10.1155/2021/7711056

**Published:** 2021-11-18

**Authors:** Zhichuang Lian, Yafang Li, Wenyi Wang, Wei Ding, Zongxin Niu, Xiaohong Yang, Chao Wu

**Affiliations:** ^1^Graduate School, Xinjiang Medical University, Urumqi, China; ^2^Department of Respiratory and Critical Care Medicine, People's Hospital of Xinjiang Uygur Autonomous Region, Urumqi 830001, China

## Abstract

This study analyzed the risk factors for patients with COVID-19 developing severe illnesses and explored the value of applying the logistic model combined with ROC curve analysis to predict the risk of severe illnesses at COVID-19 patients' admissions. The clinical data of 1046 COVID-19 patients admitted to a designated hospital in a certain city from July to September 2020 were retrospectively analyzed, the clinical characteristics of the patients were collected, and a multivariate unconditional logistic regression analysis was used to determine the risk factors for severe illnesses in COVID-19 patients during hospitalization. Based on the analysis results, a prediction model for severe conditions and the ROC curve were constructed, and the predictive value of the model was assessed. Logistic regression analysis showed that age (OR = 3.257, 95% CI 10.466–18.584), complications with chronic obstructive pulmonary disease (OR = 7.337, 95% CI 0.227–87.021), cough (OR = 5517, 95% CI 0.258–65.024), and venous thrombosis (OR = 7322, 95% CI 0.278–95.020) were risk factors for COVID-19 patients developing severe conditions during hospitalization. When complications were not taken into consideration, COVID-19 patients' ages, number of diseases, and underlying diseases were risk factors influencing the development of severe illnesses. The ROC curve analysis results showed that the AUC that predicted the severity of COVID-19 patients at admission was 0.943, the optimal threshold was −3.24, and the specificity was 0.824, while the sensitivity was 0.827. The changes in the condition of severe COVID-19 patients are related to many factors such as age, clinical symptoms, and underlying diseases. This study has a certain value in predicting COVID-19 patients that develop from mild to severe conditions, and this prediction model is a useful tool in the quick prediction of the changes in patients' conditions and providing early intervention for those with risk factors.

## 1. Introduction

The new coronavirus pneumonia (COVID-19) is an acute respiratory infectious disease caused by the new type of coronavirus (SARS-CoV-2) [1, 2]. The disease occurs insidiously and develops rapidly. In severe cases, it can quickly develop into acute respiratory distress syndrome (ARDS), septic shock, metabolic acidosis that is difficult to correct, coagulation dysfunction, etc. [3]. Therefore, it is very important to know the risk factors for COVID-19 that develops from mild to severe, identify those potentially severe cases early, and give active intervention, in order to improve the survival rate of patients. Its clinical classification [4] mainly includes the mild type (with mild clinical symptoms, no pneumonia on imaging), common type (with clinical symptoms such as fever and respiratory diseases and with findings of pneumonia on imaging), severe type, and critical type; asymptomatic carriers [5, 6] refer to those individuals who have no clinical symptoms (such as fever, cough, and sore throat) but have positive pathogenic or serum specific IgM antibodies in the respiratory tract or other origin samples, and asymptomatic carriers can also be sources of infection.

At present, the early warning model for severe/critical type of COVID-19 is mainly based on laboratory test indicators, and the predictive value of clinical symptoms for warning signs of severe COVID-19 is less researched. For example, the study by Luo et al. [7] included many laboratory indicators (routine blood tests, liver and kidney function, etc.) in the prediction model for COVID-19, while fewer clinical symptom indicators were included, which did not predict COVID-19 at earlier phases; therefore, the clinical application value of that study is limited. Studies from Poggiali and others [8] found that the predictive model using age, LDH, and CD4+ ([age × LDH]/CD4) showed 81% sensitivity and 93% specificity for early prediction. Both the sensitivity and specificity of this predictive model were not high, and the predictive evaluation could not be made at patients' admission. Therefore, its clinical predictive value needs to be evaluated. A multicenter study conducted in China [9] was developed and internally verified a nomogram predicting COVID-19 based on the symptoms, vital signs, and comorbidities of 366 laboratory-confirmed COVID-19 patients in the emergency departments. The Harrel concordance index (C-Index) of this model was 0.863 (95% CI, 0.801–0.925). Although the AUC was high and a 95% CI was sufficient, there were a few severe cases, which led to a certain deviation in the prediction results. Therefore, it is important to develop new methods to evaluate at early stages which cases may become clinically severe.

This study was performed in a designated hospital for COVID-19 in one city. It retrospectively collected the clinical data of all the 1,046 cases of confirmed and asymptomatic COVID-19 patients in this hospital from July to September in 2020, analyzed the related risk factors of developing severe illnesses, and explored the predictive value of clinical symptoms in the change from mild COVID-19 to severe conditions. This study may help in predicting the severity and prognosis of COVID-19, thereby providing early interventions for those with risk factors.

## 2. Data and Methods

### 2.1. Subjects

Data from all the 1,046 adult patients with confirmed or asymptomatic COVID-19 from July to September in 2020 in a designated hospital were retrospectively collected, and their diagnoses and classifications were in accordance with the Chinese Clinical Guidance for COVID-19 Pneumonia Diagnosis and Treatment (7th Edition) [4]. All subjects were grouped into the nonsevere group or the severe group: the former included asymptomatic, mild, and common COVID-19 patients, while the latter included the severe and critical ones.

### 2.2. Ethics

This study has been approved by the Medical Ethics Committee of the Xinjiang Uiger Municipal People's Hospital (Approval number: 2020056). Written informed consent was obtained from each participant.

### 2.3. Methods

The clinical data of 1046 COVID-19 patients were retrospectively analyzed. According to the needs of this research, the total included 25 clinical characteristic indicators were as follows: sex, age, fever, cough, fatigue, gastrointestinal symptoms, upper respiratory tract symptoms, muscle aches, headache, chest tightness, hypertension, chronic kidney disease, diabetes, coronary heart disease, cerebrovascular disease, chronic obstructive pulmonary disease (COPD), bronchial asthma, tumor, chronic hepatitis, immune deficiency, venous thrombosis, pneumothorax, acute liver injury, acute kidney injury, and electrolyte imbalance. First, the *t*-test was used to analyze the age difference between the groups, and the *chi-square* test was used to analyze the differences between the remaining indicators. Then, the selected indicators were further screened by multivariate unconditional logistic regression, and the factors affecting the severity of COVID-19 patients were analyzed and further classified and modeled. Finally, the predictive value of the model was evaluated through indicators such as receiver operating characteristic **(**ROC) curve, AUC value, sensitivity, and specificity. Based on this, a model for predicting COVID-19 patients developing severe conditions at admission was constructed.

### 2.4. Statistical Analysis

Statistical analysis was performed using SPSS 25.0 software. Quantitative data were presented as $\bar{x}\pm s$, and comparisons between groups were made by *t*-test, while qualitative data or categorical variables were expressed by the number of cases (proportion) and processed by *chi-square* test. The analysis of the risk predictive factors of COVID-19 developing into severe conditions was performed by binary logistic regression analysis, and ROC curves were plotted. *P* < 0.05 was considered a statistically significant difference.

## 3. Results

### 3.1. Baseline Data

#### 3.1.1. Characteristics of the Age Distribution of Different Types of COVID-19

This study collected 1,046 asymptomatic and confirmed COVID-19 patients, including 226 asymptomatic carriers (21.6%), with an average age of 24.56 ± 14.83 years, and 820 confirmed cases (78.4%), with an average age of 37.67 ± 18.34 years. Among the confirmed subjects, 56 (5.4%) were of a severe type and aged 63.18 ± 12.2 years, 529 (50.6%) were of common type and aged 52.02 ± 15.62 years, and 235 (20.7%) were of a mild type and aged 27.24 ± 15.88)) years. There was no significant difference in either age or sex between the two groups (*P* > 0.05).

#### 3.1.2. Symptomatic Characteristics of Confirmed COVID-19 Patients

**Figure 1:**
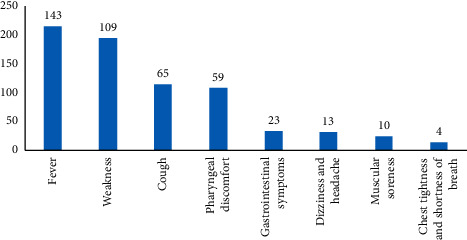
Analysis of the proportion of single symptoms in confirmed COVID-19 patients.

Among the 820 confirmed patients with COVID-19, 568 (69.27%) had clinical manifestations such as fever, fatigue, and cough, and 426 (75%) had single symptoms, of whom 143 (33.57%) had a fever, 109 (25.59%) had asthenia, 65 (15.26%) had a cough, 59 (10.52%) had a sore throat, 23 (3.15%) had nausea, 13 (2.87%) had a headache, 10 (2.29%) had muscle aches, and 4 (1.24%) had shortness of breath (Figure 1). There were 142 cases (25%) with 2 or more clinical symptoms, including 89 cases (62.68%) with 2 symptoms, 41 cases (28.87%) with 3 symptoms, and 1 case with the most symptoms of 6.

#### 3.1.3. Underlying Conditions of COVID-19 Patients

**Figure 2:**
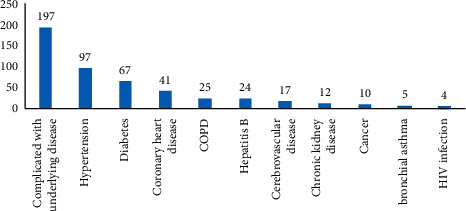
Underlying diseases in COVID-19 patients.

There were 197 cases among the 1046 COVID-19 patients (18.83%) in this study that had underlying diseases, including 97 (9.27%) with hypertension, 67 (6.41%) with diabetes, 41 (3.92%) with coronary heart disease, 25 (2.39%) with COPD, 24 (2.29%) with hepatitis B, 17 (1.63%) with cerebrovascular disease, 12 (1.15%) with chronic kidney disease, 10 (0.96%) with malignant tumor, 5 (0.48%) with bronchial asthma, and 4 (0.38%) with HIV infection (Figure 2).

**Figure 3:**
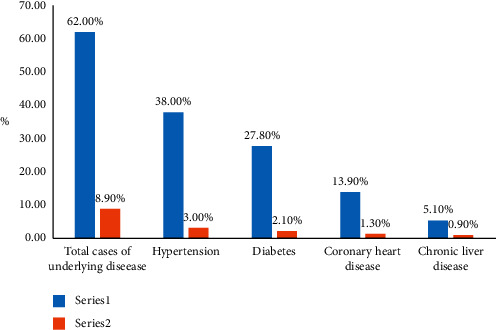
Underlying diseases in confirmed COVID-19 patients of different severities (62% of the severe and critical cases had underlying diseases, compared to 8.9% of the mild and common cases).

Of the severe and critically severe patients, 62% had complications with underlying diseases, which was higher than those of the mild and moderate cases (Figure 3).

#### 3.1.4. Clinical Characteristics of Severe and Critical COVID-19 Patients

1. *Advanced Age*. The average age of patients with severe cases was 63.18 ± 18.21 years, with a median age of 66 years, and the average age of patients with critical cases was 66.37 ± 13.69 years, with a median age of 68 years, which were significantly higher than those in other types of cases. Among the 75 severe and critical cases, 26 were 70–80 years old (accounting for 34.67%), 7 were over 80 years old (accounting for 9.33%), and cases over 70 years old accounted for 44% of the severe and critical cases. These proportions were significantly higher than those in other types of cases.
2. *With More Underlying Diseases*. Among the 56 severe cases, 38 (67.86%) had underlying diseases, and 14 (73.68%) of the 19 critically ill patients had underlying diseases. The main underlying diseases were hypertension, coronary heart disease, diabetes, chronic liver disease, chronic kidney disease, and chronic respiratory diseases. These proportions were significantly higher than those in other types of cases.

#### 3.1.5. Severe Organic Dysfunction and Complications in Severe and Critical COVID-19 Patients

During the diagnosis and treatment of 1046 cases, 105 (10.04%) developed hypokalemia, 39 (3.73%) developed an acute liver injury, 17 (1.63%) developed an acute kidney injury, 5 (0.48%) developed venous thrombosis, and 4 (0.38%) developed pneumothorax.

A respiratory failure occurred in both severe and critical cases; 49 cases with severe or critical COVID-19 (65.33%) had hypokalemia, 22 cases (29.33%) had an abnormal liver function, 6 cases (8.00%) had an abnormal renal function, 5 cases (6.67%) developed venous thrombosis, and 4 cases (5.33%) had a pneumothorax.

### 3.2. Analysis of Characteristic Clinical Indicators of the Patients in the Severe and Nonsevere Groups at Admission

A total of 25 indicators were included. Compared with nonsevere patients, severe COVID-19 patients showed significant differences (*P* < 0.05) in 17 items, including age, fever, cough, fatigue, digestive tract symptoms, chest tightness, hypertension, chronic kidney disease, diabetes, cerebrovascular disease, COPD, tumor, venous thrombosis, pneumothorax, acute liver injury, and electrolyte imbalance (Table 1).

### 3.3. Analysis of Predictors for COVID-19 Patients Developing Severe Illnesses

Logistic regression analysis was employed, the above 17 indicators with differences were included in the study, and the correlation between the above indicators and the occurrence of severe illnesses in the two groups was analyzed. The results showed that age, cough, COPD, and venous thrombosis of COVID-19 patients were the influencing factors for developing severe illnesses, and *P* < 0.05 indicated that the difference was statistically significant (Table 2).

The incidence of COVID-19 patients with an advanced age to develop severe illnesses was 1.123 times that of nonadvanced aged patients; the incidence of COVID-19 patients with a cough to develop serious illnesses was 5.088 times that of patients without cough; the incidence of COVID-19 patients with COPD to develop serious illnesses was 7.018 times that of non-COPD patients; the incidence of patients with venous thrombosis to develop serious illnesses was 162.753 times that of those without venous thrombosis.

Since Logit (1) regression analysis may eliminate meaningless indicators, to avoid errors, after the aforementioned 17 indicators with significant differences were classified into 4 dimensions and each indicator was assigned 1 point, the analysis was carried out. The four dimensions were age, clinical symptoms (fever, cough, fatigue, gastrointestinal symptoms, and chest tightness), underlying diseases (hypertension, chronic kidney disease, diabetes, cerebrovascular disease, COPD, and tumor), and complications (venous thrombosis, pneumothorax, acute liver injury, and electrolyte imbalance). Univariate analysis found that age, complications, underlying diseases, and the number of symptoms were significantly different (*P* < 0.05) (Table 3). Further logistic regression analysis found that the age of COVID-19 patients (OR = 1.115, *P* < 0.001), number of complications (OR = 2.034, *P*=0.010), number of underlying diseases (OR = 1.623, *P*=0.012), and number of symptoms (OR = 2.206, *P* < 0.001) were risk factors influencing the development of severe illnesses. *P* < 0.05 indicated a statistical significance (Table 4).

The incidence of COVID-19 patients with an advanced age to develop severe illnesses was 1.115 times that of nonadvanced aged patients (95% CI, 1.090–1.141); the incidence of COVID-19 patients with more complications to develop serious illnesses was 2.034 times that of patients with less complications (95% CI, 1.186–3.488); the incidence of COVID-19 patients with more underlying diseases to develop serious illnesses was 1.623 times that of those with less underlying diseases (95% CI, 1.111–2.373); the incidence of patients with more clinical symptoms to develop serious illnesses was 2.206 times that of those with less symptoms (95% CI, 1.542–3.155).

### 3.4. Analysis of the ROC Curve for Predicting Risk Factors for Severe COVID-19 Patients

In the single prediction, the area under the curve (AUC) of patients with severe COVID-19 was 0.910, the cutoff value (cutoff) was −8.329, the sensitivity of diagnosing severe COVID-19 was 92%, and the specificity was 79.4% (Table 5).

In the classification prediction, the AUC of patients with severe COVID-19 was 0.944, the cutoff was −2.2255, the sensitivity of diagnosing severe COVID-19 was 86.7%, and the specificity was 90% (Table 5).

**Figure 4:**
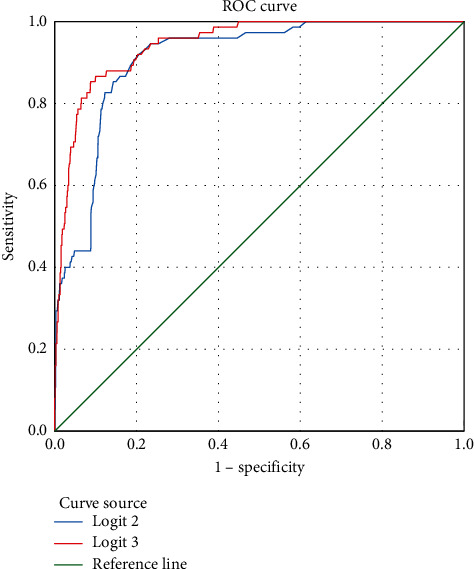
ROC curve analysis of risk factors predicting severe COVID-19.

Both AUC values were greater than 0.9, indicating high accuracy. Logistic 1 showed higher sensitivity, and Logistic 2 showed higher specificity (Figure 4). The AUC of Logistic 2 was greater than that of Logistic 1, indicating that the Logistic 2 ROC curve had more diagnostic value (Table 5).

### 3.5. Analysis of Predictors of COVID-19 Patients Developing Severe Illnesses at Admission

#### 3.5.1. Predictors of COVID-19 Patients Developing Severe Illnesses at Admission

Since complications are late symptoms of COVID-19, the aforementioned prediction model may have delayed prediction. To achieve timely and early prediction of a patient's severe risk at admission, after complications were removed, univariate analysis was employed to screen factors with differences (Table 6). Then, logistic regression analysis was applied to analyze the correlation between the above indicators and developing severe illnesses. The results showed that the age, number of symptoms, and underlying diseases of COVID-19 patients were the influencing risk factors for developing severe illnesses (Table 7). *P* < 0.05 indicated a statistically significant difference.

#### 3.5.2. Predictors of COVID-19 Patients Developing Severe Illnesses at Admission

Since Logit (3) regression analysis may eliminate meaningless indicators, to avoid errors, after eliminating the complication factor among the aforementioned 17 indicators with a significant difference, the remaining were classified into 3 dimensions, each indicator was assigned 1 point, and the analysis was carried out. The 3 dimensions were age, clinical symptoms (fever, cough, fatigue, gastrointestinal symptoms, and chest tightness), and underlying diseases (hypertension, chronic kidney disease, diabetes, cerebrovascular disease, COPD, and tumor). Univariate analysis found that age, underlying diseases, and the number of symptoms were significantly different (*P* < 0.05) (Table 8). Further logistic regression analysis found that the age of the COVID-19 patients (OR = 1.120, *P* < 0.001), underlying diseases (OR = 1.579, *P*=0.020), and the number of symptoms (OR = 2.268, *P* < 0.001) were risk factors influencing the development of severe illnesses. *P* < 0.05 indicated a statistical significance (Table 9).

#### 3.5.3. Analysis of the ROC Curve for Predicting COVID-19 Patients Developing Severe Illnesses

**Figure 5:**
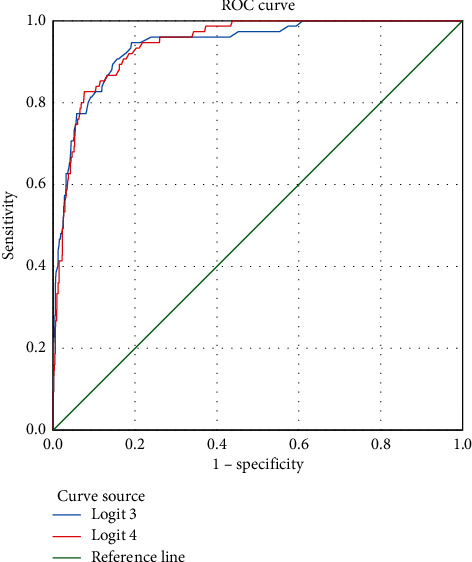
ROC curve of risk factors for predicting COVID-19 patients developing severe illnesses.

Before removing the complications factor, the AUC for predicting COVID-19 patients developing severe illnesses was 0.940, the cutoff was −3.24, the sensitivity of diagnosing the severity of COVID-19 was 94.7%, and the specificity was 80.8% (Figure 5).

After removing the complications factor, the AUC for predicting COVID-19 patients developing severe illnesses was 0.943, and the cutoff was −1.9465. The sensitivity of diagnosing the severity of COVID-19 was 82.7%, and the specificity was 92.4% (Figure 5).

Both AUC values were greater than 0.9, indicating that the accuracy was very high. The AUC of the Logistic 4 ROC curve was greater than that of the Logistic 3 ROC curve, meaning that the Logistic 4 ROC curve had more diagnostic value. The Logistic 4 ROC curve had the highest prediction specificity (Table 10).

### 3.6. Risk Prediction Model for Patients with COVID-19 Developing Severe Illnesses

Based on the above research, we established a risk prediction model for patients with COVID-19 developing severe illnesses at admission. This model has a certain value in predicting the transition of COVID-19 patients from mild conditions to severe illnesses and can be used as an indicator for the rapid prediction of changes in COVID-19 patients' conditions and administering early interventions. The link for this prediction model is https://www.healthy.vip/mode/005/index.html.

## 4. Discussion

In this study, according to the clinical data of 1046 patients with COVID-19, we developed a risk prediction model based on the age, clinical symptoms, and underlying diseases of newly admitted patients with COVID-19 to predict their development into severe conditions and further programmed a predictor on the Internet. The study screened 25 clinical factors and established a ROC regression model with an AUC score of 0.943. Clinicians can use the predictor on the web to assess the risk of newly admitted patients with COVID-19 at their admission and predict the trend of their condition to provide early intervention and treatment for those with a high risk for the occurrence of severe illnesses. Since the proportion of critically ill patients directly determines patient mortality and the cost and difficulty of medical treatment, recognizing risk and intervening early has important clinical significance for the treatment and improvement of the prognosis of patients with COVID-19 [10]. In areas with more COVID-19 patients but limited medical resources, for patients with lower risks of developing severe illnesses, early interventions can be implemented accordingly, while patients with higher risks can be provided with active diagnosis and treatment to the greatest extent. How to formulate an objective, accurate, simple, and easy-to-obtain early warning model has a certain reference value for guiding clinical work.

In some studies, the clinical symptoms of fever, cough, chest tightness, fatigue, and gastrointestinal symptoms and the underlying diseases of hypertension, COPD, chronic kidney disease, diabetes, cerebrovascular disease, and malignant tumors are important factors affecting COVID-19 development into severe illnesses [11–13]. These findings are similar to the results of this study. In this study, we found that the average age of the patients with common COVID-19 cases was 52.02 ± 15.62 years, while the average age of patients with severe cases was 63.18 ± 12.21 years. Multivariate analysis showed that age was a risk factor for patients with the common type of COVID-19 for developing severe conditions (OR = 3.257, 95% CI 1.095～1.145). This may be related to the decline in physical function, poor body resistance, and chronic diseases in the elderly. This is consistent with the findings from Zhang et al. [14, 15]. Meanwhile, the decline in ACE2 expression with age is related to the higher morbidity and mortality of older people [10].

Fever is the most common clinical manifestation of COVID-19. The peak and duration of fever in severe/critically ill patients are higher than those in common type patients. This finding may be related to the continuous stimulation of the hypothalamus by endogenous pyrogenic substances, which is the same as the characteristics of SARS patients in 2003. The body temperature of the common type of COVID-19 patients gradually decreases after treatment. When a patient continues to have a high fever after receiving active treatment (the highest body temperature exceeds 39.0°C for 2 consecutive days), we should be vigilant to whether it has a tendency to develop severe disease or has progressed to a critical condition [16]. The principal early clinical manifestations of COVID-19 in patients are fever, cough, and fatigue, which often mislead a patient to think they have a common cold and therefore lead to a delay in patients seeking medical attention and the rapid development of the disease. The clinical symptoms of severe COVID-19 patients are mainly fatigue and chest tightness, and the incubation period of severe patients is longer than that of common patients. These results are consistent with the results from Wang et al. [17]. In this study, patients with COVID-19 who had gastrointestinal symptoms had a higher chance of developing severe illnesses. This may be related to the expression of the receptors of the COVID-19 virus in almost all tissues of the body, especially in the gastrointestinal tract. Ignorance of gastrointestinal symptoms may hinder the diagnosis of some patients and exacerbate the effects of the virus through fecal or oral transmission [18–20].

In this study, 62% of severe and critical COVID-19 cases had underlying diseases, which was significantly higher than the 8.9% of mild and common cases with underlying diseases. In severe and critical cases, the proportion of patients with coronary heart disease or hypertension complications was significantly higher than that of patients with other chronic diseases. The results of multivariate analysis showed that COVID-19 complicated with coronary heart disease or hypertension was an independent risk factor for developing severe and critical illnesses. This may be related to the widespread expression of ACE2 receptors in the cardiovascular system, and patients with coronary heart disease or hypertension complications have a high expression of ACE2 [21]. Patients with coronary heart disease have poor cardiac reserve function due to myocardial ischemia or necrosis. Once they develop COVID-19, they are prone to ARDS and hypoxemia, their body's tolerance becomes low, and cardiac insufficiency is more likely to occur, leading to a sharp decrease in their condition [22]. Yang et al. [11] found that some COVID-19 patients with coronary heart disease complications had serious conditions and extremely high mortality, which is consistent with the results of our study. Studies [11, 23] have shown that ACE2, the key receptor of the new coronavirus, is expressed at a high level in the human kidney (nearly 100 times higher than that of the lungs), and ACE2 is one of the main receptors that mediate the entry of the new coronavirus into human cells. The new coronavirus can enter renal tubular cells by binding to ACE2, causing cytotoxicity and abnormal renal function. In patients with chronic kidney disease, this process has been strengthened and further aggravated the transformation of common COVID-19 to severe COVID-19. COVID-19 has a long incubation period and insidious onset. When it occurs in patients with COPD, the clinical manifestations may be more atypical and may be confused with the existing symptoms of COPD. Patients with COPD have poor basic lung function and poor tolerance to hypoxia. Once infected with COVID-19, lung function may deteriorate rapidly, and respiratory failure may easily occur [24–26]. In this study, the proportion of severe and critical COVID-19 patients with diabetes was 27.8%, which was much higher than 6.41% of patients with the common type of COVID-19 who had diabetes. This may be related to the fact that high blood sugar can inhibit the release of interleukin-10 from lymphocytes and macrophages and weaken the flow, chemotaxis, and phagocytosis of polymorphonuclear leukocytes, which further leads to disorder of the humoral immune response [17]. Patients with malignant tumors experience physical weakness and are susceptible to COVID-19. After getting COVID-19, they are more likely to develop severe cases. After receiving immunosuppressive agents, surgery, radiotherapy, or chemotherapy, the body's ability to resist pathogens may be weaker and more likely to develop severe cases of COVID-19. One study suggested that infection with SARS-CoV-2 reduced the expression and destroyed the function of ACE2, which increased the patient's blood pressure and the risk of stroke [27]. For patients with underlying cerebrovascular disease who are infected with the coronavirus, the risk of stroke will be further aggravated, and for patients with the common type, the risk of developing a severe case of COVID-19 will be increased.

After patients with COVID-19 are admitted to the hospital, their risk of developing severe or critical illness can be further judged according to their complications, the treatment plan can be adjusted in advance, and early intervention can be given if necessary to reduce the incidence of severe cases. In this study, 29.33% of severe and critically ill COVID-19 patients suffered from abnormal liver function, which was higher than the 3.73% in common cases. This liver damage may be caused by the hypoxic state or shock in COVID-19 patients, which may cause liver ischemia, hypoxia, and hypoperfusion. Studies by Sun Dawei and others [28–30] found that the incidence of liver damage in COVID-19 patients was 56.9%, among which the incidence in critically ill patients was as high as 90.9%, meaning that the incidence of liver damage in COVID-19 patients was higher, which is similar to the results of this study. Zhou et al. [21] found that 30% of COVID-19 patients have shortened prothrombin times, and coagulation dysfunction can lead to thrombosis on the surface of intravenous plaques and induce venous thrombosis, myocardial infarction, and other cardiovascular diseases. When there is venous thrombosis, a clot can easily fall off and form an embolus, which further leads to the occurrence of myocardial infarction or other severe or critical complications.

Multivariate logistic regression analysis in this study showed that age, clinical symptoms (fever, cough, fatigue, gastrointestinal symptoms, and chest tightness), underlying diseases (hypertension, chronic kidney disease, diabetes, cerebrovascular disease, COPD, and tumors), and complications (venous thrombosis, pneumothorax, acute liver injury, and electrolyte imbalance) are risk factors for severe/critical COVID-19. We further used the above factors as indicators of the early warning model through four logistic regression models and the assignment of the OR value of each risk factor. We compared them before and after admission and with or without complications as predictors and more accurately and comprehensively evaluated the value of each risk factor in the development of critical illness. The ROC curve analysis for all the above factors showed that the AUC of this early warning model reached 0.944, and when −2.2255 was used as the critical value, the sensitivity and specificity reached 86.7% and 90.0%, respectively. The efficacy of this early warning model is at a relatively high level in predicting severe/critical COVID-19. To predict the probability of severe/critical illness in patients with COVID-19 as early as possible, we removed complications after admission. Then, we analyzed the ROC curve again based on age, clinical symptoms, and underlying diseases at admission and established the prediction model. Its AUC reached 0.943, and when 1.9465 was used as the critical value, the sensitivity and specificity reached 82.7% and 92.4%, respectively, and the effectiveness of the prediction model was also at a relatively high level.

This study was based on the screening of 25 clinical characteristics of 1046 patients with COVID-19 in China. We found 3 risk factors that affect the development of severe illnesses, which can predict the possibility of COVID-19 patients developing critical illnesses at admission and can serve for triage at the patient's visit, improving the efficiency of medical resource allocation. The prediction model can be used to evaluate the risk for COVID-19 patients to progress to severe conditions.

Statistics found that approximately 20% of COVID-19 patients are seriously ill, and 5%–10% of them are transferred to the ICU and need to be intubated or to use a ventilator. Currently, in areas with scarce medical resources, it is particularly important to identify the risk for COVID-19 patients becoming critically ill in the early stages and strengthen the monitoring of patients with high risks in a timely manner. At present, most of the risk prediction models for severe illnesses are comprehensive predictions of clinical symptoms and laboratory test indicators. For example, the study by the team of Academician Zhong Nanshan [31] was based on 1590 patients with COVID-19 across the country, and “the HNC-LL score for predicting the severity of coronavirus disease 2019” was based on the team of Professor Zhu Hong [32]. Based on the general symptoms and laboratory examination outcomes of 4711 COVID-19 patients, Altschul et al. [33] proposed a model that can be used to predict the mortality of the patients during the surge period. Our model greatly differed from those reported in the literature: (1) in this model, clinical symptoms were the main indicators; (2) it was at the time of admission that this model could be used to predict the risk factors for severe disease based on the patient's age, clinical symptoms, and underlying diseases to strengthen the testing of patients and improve the utilization of medical resources. This model is easy to apply, provides individualized prediction probability for each patient, and can be used for triage during visits, greatly improving the efficiency of medical resource allocation. Early identification of patients who are at risk of becoming severely or critically ill in the early stage of the disease and timely strengthening of monitoring can help improve the prognosis of patients and have important implications for the allocation of medical resources.

This study has some limitations. First, the number of included severely ill COVID-19 patients was small, and the sample size was small. Second, the severe cases of COVID-19 in this region only account for a small part of the country's severely ill cases; therefore, this study is regional and needs to be further verified and evaluated in other parts of the country. In addition, there may be selection bias when determining the factors that affect the severity of the disease. Finally, this study was a retrospective study, so certain biases might have occurred.

## 5. Conclusions

At the admission of COVID-19 patients, we can predict the probability of severe/critical cases based on the patient's age, clinical symptoms, and underlying diseases by applying this early warning model. The indicators are relatively simple and easy to obtain and have good applicability and operability. This model can be used as a tool in clinical practice, which may help predict changes in disease conditions by dynamic scoring. It has high clinical application value and is worthy of promotion. In the future, we plan to improve this early warning model by including more research indicators, screening the indicators, and classifying each risk factor to improve its prediction accuracy.

## Figures and Tables

**Table 1:** Comparison of clinical symptom indices between common and severe COVID-19 patients at admission.

			Score	Degree of freedom	Significance

Step 0	Variate	Sex	0.557	1	0.456
		Age	201.977	1	<0.001
		Fever	31.379	1	<0.001
		Cough	57.544	1	<0.001
		Fatigue	16.094	1	<0.001
		Gastrointestinal symptoms	4.094	1	0.043
		Respiratory symptoms	0.353	1	0.553
		Muscle aches	1.175	1	0.278
		Headache	1.763	1	0.184
		Chest tightness	27.151	1	<0.001
		Hypertension	17.225	1	<0.001
		Chronic kidney disease	12.484	1	<0.001
		Diabetes	30.032	1	<0.001
		Coronary artery disease	1.619	1	0.203
		Cerebrovascular disease	6.948	1	0.008
		COPD	58.400	1	<0.001
		Bronchial asthma	1.242	1	0.265
		Tumor	7.906	1	0.005
		Chronic hepatitis	3.328	1	0.068
		Immune deficiency	1.918	1	0.166
		Venous thrombosis	40.036	1	<0.001
		Pneumothorax	38.952	1	<0.001
		Acute renal injury	2.850	1	0.091
		Acute liver injury	7.071	1	0.008
		Electrolyte imbalance	48.550	1	<0.001
	Total statistics		338.201	25	<0.001

COPD, chronic obstructive pulmonary disease.

**Table 2:** Influencing factors for COVID-19 patients developing severe illnesses.

		B	Standard error	Wald	Degree of freedom	Significance	Exp (B)	95% CI of Exp (B)	
								Lower limit	Upper limit

Step 1a	Age	0.116	0.013	83.385	1	<0.001	1.123	1.096	1.152
	Fever	0.632	0.435	2.108	1	0.147	1.881	0.802	4.415
	Cough	1.627	0.450	13.067	1	<0.001	5.088	2.106	12.292
	Fatigue	0.415	0.507	0.671	1	0.413	1.515	0.561	4.090
	Gastrointestinal symptoms	0.080	0.934	0.007	1	0.932	1.083	0.174	6.753
	Chest tightness	0.041	0.868	0.002	1	0.962	1.042	0.190	5.709
	Hypertension	−0.130	0.469	0.076	1	0.782	0.879	0.351	2.201
	Chronic kidney disease	−0.560	0.874	0.411	1	0.522	0.571	0.103	3.167
	Diabetes	0.558	0.484	1.332	1	0.248	1.747	0.677	4.509
	Cerebrovascular disease	0.797	0.983	0.657	1	0.418	2.219	0.323	15.242
	COPD	1.971	0.779	6.398	1	0.011	7.178	1.559	33.055
	Tumor	0.895	1.086	0.679	1	0.410	2.448	0.291	20.579
	Venous thrombosis	5.092	2.227	5.231	1	0.022	162.753	2.071	12787.552
	Pneumothorax	19.951	20619.154	<0.001	1	0.999	461834480.538	<0.001	.
	Acute liver injury	0.511	0.630	0.658	1	0.417	1.667	0.485	5.733
	Electrolyte imbalance	0.418	0.381	1.206	1	0.272	1.519	0.720	3.204
	Constant	−9.153	0.781	137.240	1	<0.001	<0.001		

Regression equation: Logit(1)=0.016^*∗*^Age+1.627^*∗*^Cough+1.971^*∗*^COPD+5.902^*∗*^Venous thrombosis − 9.153.

**Table 3:** Analysis of clinical symptoms in common and severe types of COVID-19 in classification analysis.

			Score	Degree of freedom	Significance

Step 0	Variate	Age	201.977	1	<0.001
		Complications	77.084	1	<0.001
		Underlying diseases	62.608	1	<0.001
		Number of symptoms	88.775	1	<0.001
	Total statistics		268.215	4	<0.001

**Table 4:** Influencing factors for COVID-19 patients to develop severe illnesses in classification analysis.

		B	Standard error	Wald	Degree of freedom	Significance	Exp (B)	95% CI of Exp (B)	
								Lower limit	Upper limit

Step 1^a^	Age	0.109	0.012	89.053	1	<0.001	1.115	1.090	1.141
	Complications	0.710	0.275	6.662	1	0.010	2.034	1.186	3.488
	Underlying diseases	0.485	0.194	6.265	1	0.012	1.623	1.111	2.373
	Number of symptoms	0.791	0.183	18.755	1	<0.001	2.206	1.542	3.155
	Constant	−8.818	0.720	150.063	1	<0.001	<0.001		

Regression equation: Logit(2)=0.109^*∗*^Age+0.710^*∗*^Number of complications+0.485^*∗*^Number of underlying diseases+0.791^*∗*^Number of symptoms − 8.818.

**Table 5:** Index analysis of Logit (1) and Logit (2).

Indices	Logistic 1 ROC curve	Logistic 2 ROC curve

AUC	0.910	0.944
Youden index	0.714	0.767
Sensitivity	0.92	0.867
Specificity	0.794	0.90
Cutoff	−8.329	−2.2255

**Table 6:** Comparison of clinical symptom indices between common and severe COVID-19 patients after removing complications.

			Score	Degree of freedom	Significance

Step 0	Variate	Age	201.977	1	<0.001
		Fever	31.379	1	<0.001
		Cough	57.544	1	<0.001
		Fatigue	16.094	1	<0.001
		Gastrointestinal symptoms	4.094	1	0.043
		Chest tightness	27.151	1	<0.001
		Hypertension	17.225	1	<0.001
		Chronic kidney disease	12.484	1	<0.001
		Diabetes	30.032	1	<0.001
		Cerebrovascular disease	6.948	1	0.008
		COPD	58.400	1	<0.001
		Tumor	7.906	1	0.005
	Total statistics		289.142	12	<0.001

Regression equation: Logit(3)0.119^*∗*^Age+1.807^*∗*^Cough+2.017^*∗*^COPD − 9.082.

**Table 7:** Influential factors of aggravation to severe COVID-19 after removing complications.

		B	Standard error	Wald	Degree of freedom	Significance	Exp (B)	95% CI of Exp (B)	
								Lower limit	Upper limit

Step 1^a^	Age	0.119	0.012	93.249	1	<0.001	1.126	1.099	1.153
	Fever	0.456	0.424	1.156	1	0.282	1.577	0.687	3.622
	Cough	1.807	0.421	18.389	1	<0.001	6.092	2.667	13.915
	Fatigue	0.390	0.499	0.611	1	0.434	1.477	0.556	3.927
	Gastrointestinal symptoms	0.023	0.886	0.001	1	0.979	1.024	0.180	5.816
	Chest tightness	0.247	0.810	0.093	1	0.760	1.281	0.262	6.268
	Hypertension	−0.153	0.461	0.110	1	0.740	0.858	0.348	2.117
	Chronic kidney disease	−0.044	0.838	0.003	1	0.958	0.957	0.185	4.946
	Diabetes	0.436	0.484	0.813	1	0.367	1.547	0.599	3.993
	Cerebrovascular disease	0.931	0.995	0.875	1	0.350	2.536	0.361	17.815
	COPD	2.017	0.792	6.480	1	0.011	7.513	1.590	35.491
	Tumor	0.731	1.101	0.441	1	0.507	2.078	0.240	17.998
	Constant	−9.082	0.755	144.843	1	<0.001	<0.001		

**Table 8:** Comparison of factors affecting the COVID-19 patients developing severe illnesses after assignment.

			Score	Degree of freedom	Significance

Step 0	Variate	Age	201.977	1	<0.001
		Underlying diseases	62.608	1	<0.001
		Number of symptoms	88.775	1	<0.001
	Total statistics		253.824	3	<0.001

**Table 9:** Factors affecting the COVID-19 patients developing severe illnesses after assignment.

		B	Standard error	Wald	Degree of freedom	Significance	Exp (B)	95% CI of Exp (B)	
								Lower limit	Upper limit

Step 1^a^	Age	0.113	0.011	98.200	1	<0.001	1.120	1.095	1.145
	Underlying diseases	0.457	0.196	5.447	1	0.020	1.579	1.076	2.318
	Number of symptoms	0.819	0.181	20.505	1	<0.001	2.268	1.591	3.232
	Constant	−8.811	0.708	154.838	1	<0.001	<0.001		

Logit(4)=0.113^*∗*^Age+0.457^*∗*^Number of underlying diseases+0.819^*∗*^Number of symptoms − 8.811.

**Table 10:** Index analysis of Logit (3) and Logit (4).

Indices	Logistic 3 ROC curve	Logistic 4 ROC curve

AUC	0.940	0.943
Youden index	0.755	0.751
Sensitivity	0.947	0.827
Specificity	0.808	0.924
Cutoff	−3.24	−1.9465

## Data Availability

The data used for the analysis in this study are available on reasonable request from the corresponding authors.
